# Prevalence and Risk of Anemia in People With Multiple Sclerosis (MS): A Systematic Review and Meta‐Analysis

**DOI:** 10.1155/anem/1374218

**Published:** 2026-08-26

**Authors:** Vasileios Giannopapas, Evangelia-Makrina Dimitriadou, Alexandra Akrivaki, Dimitrios K. Kitsos, Konstantina Stavrogianni, Athanasios K. Chasiotis, Konstantinos Tsamis, George P. Paraskevas, Maria Kosmidou, John S. Tzartos, Georgios Tsivgoulis, Sotirios Giannopoulos

**Affiliations:** ^1^ Second Department of Neurology, School of Medicine, National and Kapodistrian University of Athens, Athens, Greece, uoa.gr; ^2^ Department of Physiology, Faculty of Medicine, School of Health Sciences, University of Ioannina, Ioannina, Greece, uoi.gr; ^3^ Third Department of Internal Medicine, Aristotle University of Thessaloniki, Thessaloniki, Greece, auth.gr

**Keywords:** anemia, comorbidities, multiple sclerosis, prevalence

## Abstract

**Introduction:**

Anemia appears as a common comorbidity in a different timing and underlying pathophysiology in people with MS (PwMS) and has been proposed as a prodrome finding in MS. This systematic review and meta‐analysis aims to fulfill the need to clarify some characteristics of anemia in PwMS.

**Methods:**

A systematic literature search was performed using the MEDLINE PubMed, Scopus, and Google Scholar databases. Eligible studies underwent quality assessment using the Robins‐E, and a meta‐analysis of proportion was performed. The risk of anemia in PwMS versus healthy controls was calculated based on a subset of case–control studies. Reliability and robustness were assessed using leave‐one‐out analysis.

**Results:**

In a pool of 143,879 PwMS across 21 studies, anemia had a pooled prevalence of 12.5% (95% CI [8.1%, 17.7%], *I* ^2^  = 99.0%, *p*_*Q*_ = 0). In a subset of five studies, the relative risk of anemia between PwMS and healthy controls was 2.9 (95% CI [1.45, 5.79], *I* ^2^ = 97.6%, *p*_*z*_  = 0.002, *p*_*Q*_ < 0.001) in favor of PwMS.

**Conclusion:**

The intersection between anemia and MS presents a complex, multifaceted clinical challenge due to several pathophysiological mechanisms and DMT‐related interactions. Further structured studies are needed to evaluate the exact underlying mechanisms and the directionality of this relationship.

## 1. Introduction

Multiple sclerosis (MS) is a chronic inflammatory demyelinating disease of the central nervous system (CNS), affecting thousands of people [1]. During the course of the disease, anemia appears as a common comorbidity in different timings and underlying pathophysiology. A characteristic of great importance in anemia is having a direct impact on everyday life among people with MS (PwMS), as it can affect common symptoms of MS patients, such as fatigue or decrease in mentation, or in specific types of anemia numbness, which creates the necessity of better specifying this coexistence [2].

Among shared clinical features within anemia and MS, fatigue is quite frequent. In anemia, fatigue results primarily from reduced oxygen delivery to peripheral tissues which can be misinterpreted as an indicator of MS progression, when in fact they may stem from an underlying hematologic disturbance [3]. Cognitive impairment is another condition where MS and anemia intersect and is strongly associated with low hemoglobin levels [4–6]. Cognitive decline in anemia may be attributed to decreased cerebral blood flow and oxygenation, which leads to hypoxia‐induced alterations in iron channel expression and neurotoxicity [7]. These overlapping symptoms underscore the need for a comprehensive and interdisciplinary approach to symptom evaluation in PwMS. Failure to consider anemia as a contributing factor may result in misdiagnosis, inappropriate therapy escalation, or underestimation of potentially reversible comorbidities.

Moreover, anemia has been proposed as a prodrome finding in MS, as it seems to preexist in many cases from the clinical onset of MS [8]. On the other hand, there are several cases where anemia presents during the course of the disease with a variety of underlying causes. These underlying causes might include drug‐induced anemia, which refers to anemia as an adverse event of disease‐modifying treatments (DMTs) such as natalizumab, alemtuzumab, and interferons. The mechanism in such cases may be through hemolytic anemia, autoimmune hemolytic anemia, or blood marrow suppression [9]. Αnemia of chronic disease is another possible mechanism for anemia in PwMS, due to the inflammatory profile of the disease. More specifically, the production of cytokines, particularly interleukin‐6 (IL‐6), can induce hepcidin expression, leading to sequestration of iron within macrophages and decreased intestinal iron absorption. This functional iron deficiency results in hypoferremia and a gradual onset of anemia [10, 11]. Finally, other deficiency anemias (B12 and folic acid) have been observed in PwMS, but their exact association with the disease and their impact on it have not been entirely defined [10, 11].

This heterogeneity in the timing of anemia onset and its underlying pathophysiology creates a need for more precise characterization of this comorbidity. As a comorbidity in MS, anemia remains insufficiently defined, despite its great impact on clinical presentation of PwMS. This systematic review and meta‐analysis aims to fulfill the need to clarify some characteristics of anemia in PwMS.

## 2. Methods

### 2.1. Standard Protocol Approval–Registration

The predefined protocol for this systematic review and meta‐analysis is registered in the Open Science Framework [12]. This systematic review and meta‐analysis adheres to the Preferred Reporting Items for Systematic Reviews and Meta‐Analyses (PRISMA) and was written in accordance with the Meta‐Analysis of Observational Studies in Epidemiology (MOOSE) proposal [13, 14]. Due to the nature of this study (review), no ethics board approval or written informed consent was required.

### 2.2. Data Sources–Study Selection

The systematic literature search included two major medical databases, MEDLINE PubMed and Scopus, as well as the first 200 results of Google Scholar. Two independent reviewers (V.G. and E.M.G.) searched the databases for relevant records using the following terms: “multiple sclerosis,” “anemia,” “anaemia,” “iron deficiency.” The complete search algorithms used in MEDLINE and Scopus as well as the complete study selection process are provided in the Supporting Information.

### 2.3. Quality Control, Bias Assessment, and Data Extraction

The risk of bias for relevant domains in each included study was assessed using the Risk of Bias in Non‐randomized Studies of Exposure (ROBINS‐E) tool [15]; the complete process is described in the Supporting Information (available here).

### 2.4. Outcomes

An aggregate data meta‐analysis was performed with the inclusion of the identified population‐based studies or registries and observational cohort studies.

Our predefined primary outcome measures were twofold: (i) the pooled prevalence of anemia in PwMS; and (ii) the relative risk for anemia in PwMS compared to healthy controls. Secondary outcomes of interest comprised potential associations between demographic characteristics and MS‐related characteristics and anemia in the MS population.

### 2.5. Statistical Analysis

A random‐effects meta‐analysis was conducted to estimate pooled prevalences and 95% confidence intervals for dichotomous outcomes and performed pairwise comparisons between multiple sclerosis patients and the general population using risk ratios. Heterogeneity and publication bias were formally assessed using established statistical methods. A detailed description of all statistical analyses is provided in the Supporting Information (available here) [16–19].

## 3. Results

### 3.1. Literature Search

**FIGURE 1:**
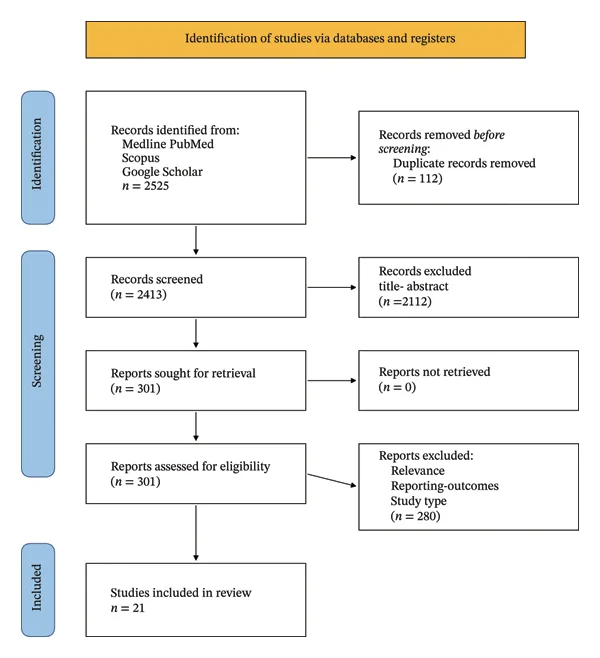
PRISMA flowchart.

The systematic literature search yielded 2525 results. After the exclusion of 112 duplicate records and the prescreening of the records based on title and abstract, 301 records were assessed. Based on the predefined inclusion–exclusion criteria, 21 records were eligible for inclusion (Figure 1).

### 3.2. Bias Assessment

Eligible articles underwent bias assessment using the Robins‐E scale. There was a low‐to‐moderate degree of bias mainly due to measurement and reporting of outcomes (E‐Figure 1).

### 3.3. Qualitative Analysis

A total of 21 studies [8, 20–39] were included in this systematic review and meta‐analysis (Table 1). The majority of the included studies reported data on anemia as a demographic variable without providing details regarding the type of anemia or any relationship with other MS‐specific and non‐MS variables. In two studies, anemia was one of the predefined outcomes. First, Koudriavtseva et al. examined the association between anemia and MS in a case–control study between PwMS and healthy controls and reported a higher rate of anemia in the MS cohort and a 2‐fold risk of MS occurrence in participants with anemia [28]. Second, Yusuf et al. 2021 also reported an increased rate of anemia in PwMS versus healthy control and additionally observed that the association of MS and anemia was higher in men than women [8]. Finally, regarding the type of anemia, only 7 studies reported a specific anemia type (2 studies reported cases of pernicious anemia, and 5 studies reported cases of deficiency anemias).

**TABLE 1:** Characteristics of included studies.

Author‐year	PwMS	Anemia cases	Age	EDSS	Disease duration	RRMS (%)	Anemia type
Edwards et al., 2004 [20]	658	6					Pernicious anemia
Buchanan et al., 2004 [21]	1309	161	57				
Kang et al., 2010 [22]	898	24					
Füvesi et al., 2010 [23]	438	17	43.3	2.6	10.1		
Marrie et al., 2010 [24]	8672	518			16.2	80.5	
Rodrigo et al., 2011 [25]	72	28	43	1.7	11	100	Deficiency anemia
Miri et al., 2013 [26]	205	73	32.8		6	87.3	
Shaygannejad et al., 2013 [27]	126	34	32.3	1.8	3.6		Deficiency anemias
Koudriavtseva et al., 2015 [28]	187	35	45.2	3.1	10.8	80.8	
Tettey et al., 2016 [29]	198	25	47.4	3	6	75.3	
Cikrikcioglu et al., 2016 [30]	243	39	37.4				Deficiency anemias
Yusuf et al., 2020 [8]	966	155	75.4				
Lo et al., 2020 [31]	1518	202	55.7		20.5	81.8	
Mendizabal et al., 2020 [32]	16,629	1504					Deficiency anemia
Chou et al., 2020 [33]	2526	210	45.03				
Afifi et al., 2021 [34]	76	38	30			100	
Bhattacharjee et al., 2021 [35]	541	28					
Maric et al., 2021 [36]	2725	109	55.8	4	21.6	62.9	Pernicious anemia
Donze et al., 2022 [37]	352	35	48.1	15.3		60.8	
Padarti et al., 2022 [38]	95,537	13,318	57				
Li et al., 2024 [39]	10,003	704	44.08				

*Note:* PwMS: people with MS; age, EDSS, and disease duration are presented by mean values, disease duration (years).

Abbreviations: EDSS, expanded disability severity scale; RRMS, relapsing remitting multiple sclerosis.

### 3.4. Quantitative Analyses

#### 3.4.1. Primary Outcomes

**FIGURE 2:**
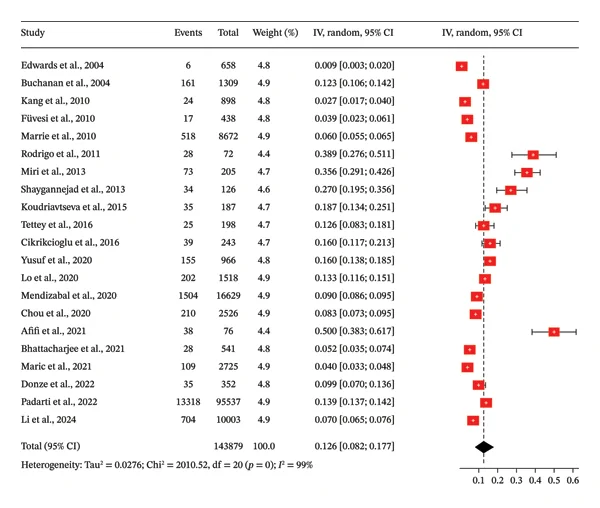
Forest plot: prevalence of anemia in PwMS. Pooled prevalence, 0.126; 95% CI [0.082, 0.177]; *I* ^2^ = 99%.

**FIGURE 3:**
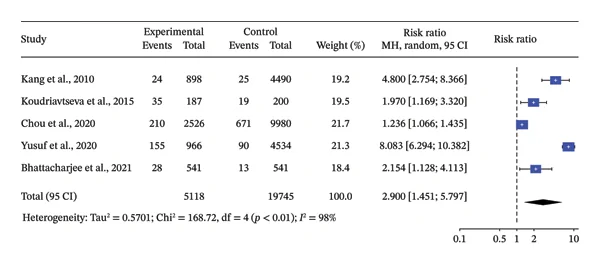
Forest plot: relative risk of anemia between PwMS and HC. RR, 2.900; 95% CI [1.451, 5.797]; *I* ^2^ = 98%.

In a pool of 143,879 PwMS, anemia had a pooled prevalence of 12.5% (95% CI [8.1%, 17.7%], *I* ^2^  = 99.0%, *p*_*Q*_  = 0) (Figure 2). Furthermore, in a subset of five studies that included healthy controls, the relative risk of anemia between PwMS and healthy controls was 2.9 (95% CI [1.45, 5.79], *I* ^2^ = 97.6%, *p*_*z*_  = 0.002, *p*_*Q*_ < 0.001) in favor of PwMS, indicating an increased risk compared to healthy controls (Figure 3).

#### 3.4.2. Secondary Outcomes

**FIGURE 4:**
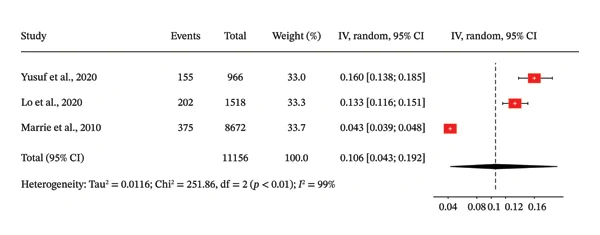
Forest plot: prevalence of anemia pre‐MS diagnosis (or at the time of diagnosis). Pooled prevalence, 0.106; 95% CI [0.043, 0.192]; *I* ^2^ = 99%.

A subset of three studies included data on the number of PwMS with anemia pre‐MS or at the time of MS diagnosis. In a pool of 11,156 PwMS, anemia was present in 10.5% (95% CI [4.2%, 19.1%], *I* ^2^ = 99.2%, *p*_*Q*_ < 0.001) pre‐MS diagnosis or at the time of diagnosis (Figure 4). Additional sensitivity analyses using regression were performed to assess the potential relationship between age, disability level, disease duration, and anemia prevalence. There was a statistically significant relationship between age and prevalence of anemia (*β* = −0.6%, *p* = 0.03), indicating a 0.6% decrease in anemia prevalence with each 1‐year increase of patient’s age. Regarding MS disease duration, even though it did not pass the statistical significance threshold, there was a statistical trend between MS disease duration and prevalence of anemia (*β* = −1%, *p* = 0.06), which indicates a 1% drop in anemia prevalence with each 1‐year increase in MS disease duration. Given the high degree of heterogeneity, a leave‐one‐out sensitivity analysis was performed to assess the robustness of the results. The sequential removal of each study resulted in pooled effects ranging from −2.21 to −1.99, with the confidence intervals remaining narrow and overlapping across all iterations. No single study meaningfully altered the magnitude or direction of the overall effect (E‐Table 1). The largest downward shifts occurred when Afifi et al. [34], Rodrigo et al. [25], or Miri et al. [26] were omitted; however, these changes were small and did not affect the meta‐analytic conclusions. Likewise, the removal of early studies such as Edwards et al. [20] or Bhattacharjee et al. [35] resulted in minimal changes to the pooled estimate. Overall, the analysis indicates that no individual study exerted disproportionate influence, supporting the stability and reliability of the meta‐analytic results.

#### 3.4.3. Publication Bias

Asymmetry in funnel plot inspection and the corresponding results of Egger’s linear regression test (*β* = −3.4, *p* = 0.21) are indicative of moderate bias without small study effects (E‐Figure 1).

## 4. Discussion

This is the first systematic review and meta‐analysis examining the prevalence of anemia in the MS population. In our study, a pool of 143,879 PwMS were included with the prevalence of anemia ranging from 8.1% to 17.7% (centering around 12.5%). When examining the relative risk of anemia in PwMS, we found that PwMS had a RR of 2.9 compared to healthy controls. Although it is uncertain whether anemia is a precursor of MS or PwMS have an increased risk of developing anemia or both, it is worth mentioning that in this meta‐analysis anemia was present in 10.9% of the participants before or at the time of disease diagnosis.

Regarding the prevalence of anemia in PwMS according to the results of the present meta‐analysis, this is approximately 12.5%, a finding close to previously reported rate from a smaller cohort of MS patients with a prevalence of anemia 18.7% [28]. This prevalence underscores that anemia is a relatively common comorbidity in PwMS and should not be underrecognized as it can potentially exacerbate clinical symptoms or present with clinical symptoms that are frequently attributed to MS. These symptoms may falsely be interpreted as disease progression or relapse, which can be interpreted into suboptimal response to DMTs, leading to misclassification of disability accumulation.

Pathophysiologically, multiple mechanisms may contribute to anemia in MS as already mentioned. Some of these mechanisms include impaired iron metabolism, nutritional deficiencies, such as folic acid and vitamin B12 deficiency, DMT‐induced anemia by causing bone marrow suppression, and autoimmune hemolytic anemia. These facts make systematic evaluation of anemia crucial and should be integrated into the assessment of clinical worsening as treating it may reduce symptom burden, specify patients’ true neurological baseline, and make accurate disease monitoring and decision making.

More specifically, chronic inflammation, an integral feature of MS, is one of the most likely contributors. It seems that the continuous inflammatory activity leads to release of proinflammatory factors such as IL‐6 and TNF‐a. These cytokines stimulate the production of hepcidin, which can lead to the reduction of the iron absorption and promote iron retention within immune cells. The above leads to the phenomenon known as “anemia of inflammation” [38]. Additionally, according to a recent study by Riedl et al. [40], during inflammatory cytokine signaling (TNF‐α and IFN‐γ), oligodendrocytes have impaired iron uptake, while myeloid cells (microglia/macrophages) accumulate iron—especially at the rims of chronic lesions. This is suggestive of impaired iron redistribution in brain tissue, which might mirror systemic effects, i.e., reduced availability of iron for erythropoiesis or functional iron deficiency even if iron stores are adequate [40]. Other deficiency anemias, for example, vitamin B12 or folic acid deficiency, due to dietary changes or gastrointestinal pathologies, may also present as a common cause of anemia. Moreover, there are several DMTs that have been proposed to increase the risk of anemia in PwMS. Natalizumab, alemtuzumab, and rarely interferons and cladribine have been reported to be responsible for the development of anemia. Alemtuzumab has been linked to autoimmune hemolytic anemia, due to its ability to induce secondary autoimmunity [41]. Natalizumab leads to anemia through bone marrow suppression, due to its effects on hematopoietic progenitor cell trafficking. Cladribine rarely causes anemia through dose‐dependent bone marrow suppression [42]. Finally, interferons have also been reported to cause anemia (the exact underlying pathophysiology has not yet been clarified). Some of the proposed mechanisms from case reports are autoimmune anemia, aplastic anemia, or microangiopathic hemolytic anemia, but current evidence is insufficient to support these scenarios [43–45].

Regarding the clinical implications that anemia can have in PwMS, previous studies have linked the presence of anemia to reduced maximal and submaximal exercise capacity with a previous meta‐analysis by Webb et al. reporting a proportional relationship between max VO2 and hemoglobin levels [46]. Furthermore, iron deficiency and low hemoglobin can impair mitochondrial function, leading to generalized weakness, reduced muscle endurance, sarcopenia [47], and increased risk of frailty and falls [48]. Finally, anemia and related nutritional deficiencies that either cause or accompany it have been linked to cognitive decline, mild cognitive impairment, and dementia in adults. This can be attributed to (a) neurobiological changes that impair cognitive function due to low levels of iron (essential for myelination, neurotransmitter synthesis, and neuronal mitochondrial function) or B12 deficiency (myelin maintenance and one‐carbon metabolism); (b) chronic brain hypoxia that may accelerate neurodegeneration; and (c) inflammation and vascular injury (in cases of anemia of inflammation) [47, 49].

Given the existence of several potential pathophysiological mechanisms and DMT‐related interaction that support the association between the two conditions, the exact nature of the relationship between MS and anemia is not yet clarified. More longitudinal and mechanistic research is necessary in order to determine whether anemia contributes to MS onset or during the course of the disease and how it could influence long‐term outcomes of the disease. Understanding all these connections could help with clinical monitoring and therapeutic decisions.

Regarding the limitations of this study, the majority of the included studies are cross‐sectional, thus not allowing causal inference. Second, there is a high degree of heterogeneity, which is likely due to variations in study design, sample size, diagnostic criteria, measurement methods, disease severity, demographic characteristics, and geographical settings and based on the results of the leave‐one‐out sensitivity analysis does not undermine the robustness and reliability of the pooled results. Finally, there was a lack of reported data regarding the order or timing of events, which prevented any analysis of directionality. As a result, it was not possible to examine cause‐and‐effect relationships or assess whether one condition may act as a precursor to the other.

## 5. Conclusions

In this systematic review and meta‐analysis, the prevalence of anemia in PwMS ranged from 8.1% to 17.7%, centering around 12.5%. These findings underscore the importance of screening for anemia from disease onset and throughout its course, given its role as a common and potentially reversible comorbidity with meaningful impact on the clinical performance of PwMS. Further research into the underlying mechanisms of anemia in this population is warranted to refine screening strategies and ultimately improve clinical outcomes.

## Funding

The authors received no funding for this work.

## Disclosure

All authors have read and approved the final version of the manuscript. The corresponding author had full access to all of the data in this study and takes complete responsibility for the integrity of the data and the accuracy of the data analysis.

## Conflicts of Interest

The authors declare no conflicts of interest.

## Supporting Information

Additional supporting information can be found online in the Supporting Information section.

## Supporting information


**Supporting Information 1** Study selection process, quality control and bias assessment process, and statistical analysis. Supporting Figure: E‐figure 1: Robins‐E traffic light plot. E‐figure 2: Funnel plot for publication bias. Supporting tables Table titles: E‐table 1: Pooled estimated from the leave‐one‐out analysis.

**Supporting Information 2** PRISMA checklist.

## Data Availability

Data are available on request from the authors.

## References

[1] Boutitah-Benyaich I. , Eixarch H. , Villacieros-Álvarez J. et al., Multiple Sclerosis: Molecular Pathogenesis and Therapeutic Intervention, Signal Transduction and Targeted Therapy. (October 2025) 10, no. 1, 10.1038/s41392-025-02415-4. PMC12488951 41034190

[2] Deleva N. S. , Multiple Sclerosis Associated With Anaemic Syndrome: A Retrospective Analysis and Literature Review, Journal of IMAB–Annual Proceeding (Scientific Papers). (June 2012) 18, no. 1, 203–205, 10.5272/jimab.2012181.203.

[3] Balducci L. , Anemia, Fatigue and Aging, Transfusion Clinique et Biologique. (December 2010) 17, no. 5–6, 375–381, 10.1016/j.tracli.2010.09.169. 21067951

[4] Agrawal S. , Kumar S. , Ingole V. et al., Does Anemia Affects Cognitive Functions in Neurologically Intact Adult Patients: Two Year Cross Sectional Study at Rural Tertiary Care Hospital, Journal of Family Medicine and Primary Care. (2019) 8, no. 9, 3005–3008, 10.4103/jfmpc.jfmpc_599_19. PMC6820398 31681682

[5] Kung W. M. , Yuan S. P. , Lin M. S. et al., Anemia and the Risk of Cognitive Impairment: An Updated Systematic Review and Meta-Analysis, Brain Sciences. (June 2021) 11, no. 6, 10.3390/brainsci11060777. PMC8231247 34208355

[6] Murray-Kolb L. E. and Beard J. L. , Iron Treatment Normalizes Cognitive Functioning in Young Women, American Journal of Clinical Nutrition. (March 2007) 85, no. 3, 778–787, 10.1093/ajcn/85.3.778. 17344500

[7] Gattas B. S. , Ibetoh C. N. , Stratulat E. et al., The Impact of Low Hemoglobin Levels on Cognitive Brain Functions, Cureus. (2020) https://www.cureus.com/articles/42910-the-impact-of-low-hemoglobin-levels-on-cognitive-brain-functions, 10.7759/cureus.11378. PMC7723430 33312780

[8] Yusuf F. L. , Wijnands J. M. , Kingwell E. et al., Fatigue, Sleep Disorders, Anaemia and Pain in the Multiple Sclerosis Prodrome, Multiple Sclerosis (Houndmills, Basingstoke, England). (February 2021) 27, no. 2, 290–302, 10.1177/1352458520908163. 32250183

[9] Scavone C. , Liguori V. , Adungba O. J. et al., Disease-Modifying Therapies and Hematological Disorders: A Systematic Review of Case Reports and Case Series, Frontiers in Neurology. (2024) 15, 10.3389/fneur.2024.1386527. PMC11217193 38957352

[10] Duarte-Silva E. , Meuth S. G. , and Peixoto C. A. , The Role of Iron Metabolism in the Pathogenesis and Treatment of Multiple Sclerosis, Frontiers in Immunology. (March 2023) 14, 10.3389/fimmu.2023.1137635. PMC10064139 37006264

[11] Poggiali E. , Migone De Amicis M. , and Motta I. , Anemia of Chronic Disease: A Unique Defect of Iron Recycling for Many Different Chronic Diseases, European Journal of Internal Medicine. (January 2014) 25, no. 1, 12–17, 10.1016/j.ejim.2013.07.011. 23988263

[12] Giannopapas V. , Prevelance of Anaemia in the Multiple Sclerosis Population, 2025, https://osf.io/me2w4/.

[13] Page M. J. , McKenzie J. E. , Bossuyt P. M. et al., The PRISMA 2020 Statement: An Updated Guideline for Reporting Systematic Reviews, BMJ. (March 2021) 71. 10.1186/s13643-021-01626-4 PMC8008539 33781348

[14] Brooke B. S. , Schwartz T. A. , and Pawlik T. M. , MOOSE Reporting Guidelines for Meta-Analyses of Observational Studies, JAMA Surgery. (August 2021) 156, no. 8, 10.1001/jamasurg.2021.0522. 33825847

[15] Higgins J. P. T. , Morgan R. L. , Rooney A. A. et al., A Tool to Assess Risk of Bias in Non-Randomized Follow-Up Studies of Exposure Effects (ROBINS-E), Environment International. (April 2024) 186, 10.1016/j.envint.2024.108602. PMC11098530 38555664

[16] Abdulmajeed J. , Chivese T. , and Doi S. A. R. , Overcoming Challenges in Prevalence Meta-Analysis: The Case for the Freeman-Tukey Transform, BMC Medical Research Methodology. (April 2025) 25, no. 1, 10.1186/s12874-025-02527-z. PMC11971753 40188320

[17] DerSimonian R. and Laird N. , Meta-Analysis in Clinical Trials Revisited, Contemporary Clinical Trials. (November 2015) 45, 139–145, 10.1016/j.cct.2015.09.002. 26343745 PMC4639420

[18] Choi G. J. and Kang H. , Heterogeneity in Meta-Analyses: An Unavoidable Challenge Worth Exploring, Korean Journal of Anesthesiology. (August 2025) 78, no. 4, 301–314, 10.4097/kja.25001. 39956636 PMC12326561

[19] Lin L. and Chu H. , Quantifying Publication Bias in Meta-Analysis, Biometrics. (September 2018) 74, no. 3, 785–794, 10.1111/biom.12817. 29141096 PMC5953768

[20] Edwards L. J. and Constantinescu C. S. , A Prospective Study of Conditions Associated With Multiple Sclerosis in a Cohort of 658 Consecutive Outpatients Attending a Multiple Sclerosis Clinic, Multiple Sclerosis Journal. (October 2004) 10, no. 5, 575–581, 10.1191/1352458504ms1087oa. 15471376

[21] Buchanan R. J. , Martin R. A. , Wang S. , and Ju H. , Analyses of Nursing Home Residents With Multiple Sclerosis at Admission and One Year After Admission, Multiple Sclerosis Journal. (February 2004) 10, no. 1, 74–79, 10.1191/1352458504ms969oa. 14760956

[22] Kang J.-H. , Chen Y.-H. , and Lin H.-C. , Comorbidities Amongst Patients With Multiple Sclerosis: A Population-Based Controlled Study, European Journal of Neurology. (September 2010) 17, no. 9, 1215–1219, 10.1111/j.1468-1331.2010.02971.x. 20192982

[23] Füvesi J. , Bencsik K. , Losonczi E. et al., Factors Influencing the Health-Related Quality of Life in Hungarian Multiple Sclerosis Patients, Journal of the Neurological Sciences. (June 2010) 293, no. 1–2, 59–64, 10.1016/j.jns.2010.03.007. 20394948

[24] Marrie R. A. , Rudick R. , Horwitz R. et al., Vascular Comorbidity is Associated With More Rapid Disability Progression in Multiple Sclerosis, Neurology. (March 2010) 74, no. 13, 1041–1047, 10.1212/wnl.0b013e3181d6b125. 20350978 PMC2848107

[25] Rodrigo L. , Hernández-Lahoz C. , Fuentes D. , Alvarez N. , López-Vázquez A. , and González S. , Prevalence of Celiac Disease in Multiple Sclerosis, BMC Neurology. (December 2011) 11, no. 1, 10.1186/1471-2377-11-31. PMC3065402 21385364

[26] Miri S. , Rohani M. , Sahraian M. A. et al., Restless Legs Syndrome in Iranian Patients With Multiple Sclerosis, Neurological Sciences. (July 2013) 34, no. 7, 1105–1108, 10.1007/s10072-012-1186-7. 22960874

[27] Shaygannejad V. , Ardestani P. E. , Ghasemi M. , and Meamar R. , Restless Legs Syndrome in Iranian Multiple Sclerosis Patients: A Case-Control Study, International Journal of Preventive Medicine. (May 2013) 4, no. Suppl 2, S189–S193. 23776722 PMC3678216

[28] Koudriavtseva T. , Renna R. , Plantone D. , Mandoj C. , Piattella M. C. , and Giannarelli D. , Association Between Anemia and Multiple Sclerosis, European Neurology. (2015) 73, no. 3–4, 233–237, 10.1159/000381212. 25823947

[29] Tettey P. , Siejka D. , Simpson Jr S. et al., Frequency of Comorbidities and Their Association With Clinical Disability and Relapse in Multiple Sclerosis, Neuroepidemiology. (2016) 46, no. 2, 106–113, 10.1159/000442203. 26784322

[30] Cikrikcioglu M. A. , Ozcan M. E. , Halac G. et al., Could Heterozygous Beta Thalassemia Provide Protection Against Multiple Sclerosis?, Medical Science Monitor. (December 2016) 22, 4854–4858, 10.12659/msm.898192. 27941710 PMC5154712

[31] Lo L. M. P. , Taylor B. V. , Winzenberg T. , Palmer A. J. , Blizzard L. , and Van Der Mei I. , Change and Onset-Type Differences in the Prevalence of Comorbidities in People With Multiple Sclerosis, Journal of Neurology. (February 2021) 268, no. 2, 602–612, 10.1007/s00415-020-10194-x. 32880720

[32] Mendizabal A. , Thibault D. P. , Crispo J. A. , Paley A. , and Willis A. W. , Comorbid Disease Drives Short-Term Hospitalization Outcomes in Patients With Multiple Sclerosis, Neurology. Clinical Practice. (June 2020) 10, no. 3, 255–264, 10.1212/cpj.0000000000000838. 32642327 PMC7292556

[33] Chou I. J. , Kuo C. F. , Tanasescu R. et al., Comorbidity in Multiple Sclerosis: Its Temporal Relationships With Disease Onset and Dose Effect on Mortality, European Journal of Neurology. (January 2020) 27, no. 1, 105–112, 10.1111/ene.14040. 31309645

[34] Afifi Z. E. , Shehata R. I. , El Sayed A. F. , Hammad E. S. M. , and Salem M. R. , Nutritional Status of Multiple Sclerosis (MS) Patients Attending Kasr Alainy MS Unit: An Exploratory Cross-Sectional Study, Journal of the Egyptian Public Health Association. (December 2021) 96, no. 1, 10.1186/s42506-021-00080-3. PMC8276896 34255211

[35] Bhattacharjee S. , Yegezu Z. , Kollecas K. , Duhrkopf K. , Hashemi L. , and Greene N. , Influence of Comorbidities on Healthcare Expenditures and Perceived Physical and Mental Health Status Among Adults With Multiple Sclerosis: A Propensity Score-Matched US National-Level Study, ClinicoEconomics and Outcomes Research. (May 2021) 13, 377–394, 10.2147/ceor.s305154. 34017188 PMC8129918

[36] Maric G. D. , Pekmezovic T. D. , Mesaros S. T. et al., The Prevalence of Comorbidities in Patients With Multiple Sclerosis: Population-Based Registry Data, Neurological Sciences. (May 2021) 42, no. 5, 1887–1893, 10.1007/s10072-020-04727-5. 32964347

[37] Donzé C. , Massot C. , Defer G. et al., NUTRISEP: Assessment of the Nutritional Status of Patients With Multiple Sclerosis and Link to Fatigue, Revue Neurologique. (April 2023) 179, no. 4, 282–288, 10.1016/j.neurol.2022.10.004. 36792421

[38] Weiss G. , Ganz T. , and Goodnough L. T. , Anemia of Inflammation, Blood. (2019) 133, no. 1, 40–50, 10.1182/blood-2018-06-856500. 30401705 PMC6536698

[39] Li J. , Hutton G. J. , Varisco T. J. , Lin Y. , Essien E. J. , and Aparasu R. R. , Factors Associated With the Initiation of High-Efficacy Disease-Modifying Agents Over Moderate-Efficacy Disease-Modifying Agents in Multiple Sclerosis, Multiple Sclerosis and Related Disorders. (November 2024) 91, 10.1016/j.msard.2024.105896. 39342811

[40] Riedl C. J. , Bormann D. , Steinmaurer A. et al., Inflammation Alters Myeloid Cell and Oligodendroglial Iron-Handling in Multiple Sclerosis, Acta Neuropathologica Communications. (June 2025) 13, no. 1, 10.1186/s40478-025-02020-0. PMC12135419 40468400

[41] Ruck T. , Bittner S. , Wiendl H. , and Meuth S. , Alemtuzumab in Multiple Sclerosis: Mechanism of Action and Beyond, International Journal of Molecular Sciences. (July 2015) 16, no. 7, 16414–16439, 10.3390/ijms160716414. 26204829 PMC4519957

[42] Langtry H. D. and Lamb H. M. , Cladribine: A Review of Its Use in Multiple Sclerosis, BioDrugs Clinical Immunotherapeutics, Biopharmaceuticals and Gene Therapy. (May 1998) 9, no. 5, 419–433, 10.2165/00063030-199809050-00006. 18020575

[43] Rieckmann P. , O’Connor P. , Francis G. S. , Wetherill G. , and Alteri E. , Haematological Effects of Interferon-Beta-1a (Rebif) Therapy in Multiple Sclerosis, Drug Safety. (2004) 27, no. 10, 745–756, 10.2165/00002018-200427100-00005. 15350158

[44] Alanoglu G. , Kilbas S. , Arslan C. , Senol A. , and Kutluhan S. , Autoimmune Hemolytic Anemia During Interferon-Beta-I B Treatment for Multiple Sclerosis, Multiple Sclerosis (Houndmills, Basingstoke, England). (June 2007) 13, no. 5, 683–685, 10.1177/1352458506071333. 17548453

[45] Aslam A. K. and Singh T. , Aplastic Anemia Associated With Interferon Beta-1a, American Journal of Therapeutics. (2002) 9, no. 6, 522–523, 10.1097/00045391-200211000-00011. 12424511

[46] Webb K. L. , Gorman E. K. , Morkeberg O. H. et al., The Relationship Between Hemoglobin and VO2max: A Systematic Review and Meta-Analysis, PLoS One. (2023) 18, no. 10. 10.1371/journal.pone.0292835 PMC10569622 37824583

[47] Wang J. , Wang C. , Li X. et al., Association of Anemia With Cognitive Function and Dementia Among Older Adults: The Role of Inflammation, Journal of Alzheimer’s Disease. (2023) 96, no. 1, 125–134, 10.3233/jad-230483. PMC10657670 37742647

[48] Guralnik J. , Ershler W. , Artz A. et al., Unexplained Anemia of Aging: Etiology, Health Consequences, and Diagnostic Criteria, Journal of the American Geriatrics Society. (March 2022) 70, no. 3, 891–899, 10.1111/jgs.17565. 34796957 PMC9298858

[49] Jáuregui-Lobera I. , Iron Deficiency and Cognitive Functions, Neuropsychiatric Disease and Treatment. (2014) 10, 2087–2095, 10.2147/ndt.s72491. 25419131 PMC4235202

