# Supplementary material for: Prevalence and Risk of Anemia in People With Multiple Sclerosis (MS): A Systematic Review and Meta‐Analysis
Source: Anemia. 2026 Aug 26;2026:1374218. doi: 10.1155/anem/1374218 (PMC13519306; doi:10.1155/anem/1374218)
Supplement: Supplementary file 1 [file ANEM-2026-1374218-s001.pdf]

PRISMA 2020 Checklist

Item #	Checklist item	Location where item is reported
**1**	Identify the report as a systematic review.	Title; Introduction
**2**	See the PRISMA 2020 for Abstracts checklist.	-
**3**	Describe the rationale for the review in the context of existing knowledge.	Introduction
**4**	Provide an explicit statement of the objective(s) or question(s) the review addresses.	Introduction
**5**	Specify the inclusion and exclusion criteria for the review and how studies were grouped for the syntheses.	Methods: Data Sources-Study selection
**6**	Specify all databases, registers, websites, organizations, reference lists and other sources searched or consulted to identify studies. Specify the date when each source was last searched or consulted.	Methods: Data Sources-Study selection
**7**	Present the full search strategies for all databases, registers and websites, including any filters and limits used.	Supplement
**8**	Specify the methods used to decide whether a study met the inclusion criteria of the review, including how many reviewers screened each record and each report retrieved, whether they worked independently, and if applicable, details of automation tools used.	Methods: Data Sources-Study selection; Supplement
**9**	Specify the methods used to collect data from reports, including how many reviewers collected data from each report, whether they worked independently, any processes for obtaining or confirming data from study investigators, and if applicable, details of automation tools used.	Methods: Quality control, bias assessment & data extraction; Supplement
**10a**	List and define all outcomes for which data were sought. Specify whether all results compatible with each outcome domain were sought, and if not, the methods used to decide which results to collect.	Methods: Outcomes; Supplement
**10b**	List and define all other variables for which data were sought (e.g. participant and intervention characteristics, funding sources). Describe any assumptions made about missing or unclear information.	Methods: Outcomes; Supplement
**11**	Specify the methods used to assess risk of bias in the included studies, including details of the tool(s) used, how many reviewers assessed each study and whether they worked independently.	Methods: Quality control, bias assessment & data extraction; Supplement
**12**	Specify for each outcome the effect measure(s) used in the synthesis or presentation of results.	Methods: Statistical analysis; Supplement
**13a**	Describe the processes used to decide which studies were eligible for each synthesis.	Methods: Statistical analysis; Supplement
**13b**	Describe any methods required to prepare the data for presentation or synthesis.	Methods: Statistical analysis; Supplement
**13c**	Describe any methods used to tabulate or visually display results of individual studies and syntheses.	n/a
**13d**	Describe any methods used to synthesize results and provide a rationale for the choice(s). If meta-analysis was performed, describe the model(s), heterogeneity assessment, and software used.	n/a
**13e**	Describe any methods used to explore possible causes of heterogeneity among study results.	Methods: Statistical analysis; Supplement
**13f**	Describe any sensitivity analyses conducted to assess robustness of the synthesized results.	Methods: Statistical analysis; Supplement
**14**	Describe any methods used to assess risk of bias due to missing results (reporting bias).	Methods: Statistical analysis; Supplement
**15**	Describe any methods used to assess certainty (or confidence) in the body of evidence for an outcome.	Methods: Statistical analysis; Supplement
**16a**	Describe the results of the search and selection process, ideally using a flow diagram.	Results: Literature search

Item #	Checklist item	Location where item is reported
**16b**	Cite studies that might appear to meet the inclusion criteria but were excluded, and explain why.	n/a
**17**	Cite each included study and present its characteristics.	Results: Qualitative analysis
**18**	Present assessments of risk of bias for each included study.	Results: Bias Assessment
**19**	For all outcomes, present summary statistics and effect estimates with precision for each study.	Results: Quantitative analyses
**20a**	For each synthesis, summarise characteristics and risk of bias among contributing studies.	Results: Qualitative analysis; Quantitative analyses
**20b**	Present results of all statistical syntheses conducted, including summary estimates and heterogeneity.	Results: Quantitative analyses
**20c**	Present results of all investigations of possible causes of heterogeneity.	Results: Quantitative analyses (Secondary outcomes)
**20d**	Present results of all sensitivity analyses conducted.	Results: Quantitative analyses (Secondary outcomes)
**21**	Present assessments of risk of bias due to missing results for each synthesis assessed.	n/a
**22**	Present assessments of certainty (or confidence) in the body of evidence for each outcome assessed.	Results: Publication bias
**23a**	Provide a general interpretation of the results in the context of other evidence.	Discussion
**23b**	Discuss any limitations of the evidence included in the review.	Discussion (Limitations)
**23c**	Discuss any limitations of the review processes used.	Discussion (Limitations)
**23d**	Discuss implications of the results for practice, policy, and future research.	Discussion (Conclusions)
**24a**	Provide registration information for the review or state that the review was not registered.	Methods: Standard protocol approval-Registration
**24b**	Indicate where the review protocol can be accessed or state that a protocol was not prepared.	Methods: Standard protocol approval-Registration
**24c**	Describe and explain any amendments to information provided at registration or in the protocol.	Methods: Standard protocol approval-Registration
**25**	Describe sources of financial or non-financial support and the role of funders or sponsors.	Declarations: Funding
**26**	Declare any competing interests of review authors.	Declarations: Competing interests
**27**	Report which materials are publicly available and where they can be found.	Declarations: Data availability
