# Supplementary material for: Prevalence and Risk of Anemia in People With Multiple Sclerosis (MS): A Systematic Review and Meta‐Analysis
Source: Anemia. 2026 Aug 26;2026:1374218. doi: 10.1155/anem/1374218 (PMC13519306; doi:10.1155/anem/1374218)
Supplement: Supplementary file 2 [file ANEM-2026-1374218-s002.docx]

E-materials (Supplement)

Study Selection Process

Quality control and Bias assessment process

Statistical Analysis

**Figure legends:**

E-figure 1: Robins-E traffic light plot

E-figure 2: Funnel plot for publication bias

**Table titles:**

E-table 1: Pooled estimated from the Leave one Out analysis

**Study Selection Process**

No language restriction was applied but the search focused on studies published after 1990. The search spanned from inception of each electronic database to May 19th, 2025.

Population-based studies or registries, and observational cohort studies reporting on anemia in PwMS were eligible for inclusion. Based on the pre-defined protocol , we excluded studies: a. including patients with diagnosis of clinically isolated syndrome (CIS); b. uncertain anemia diagnoses according to our pre-defined inclusion criteria; c. reporting outcomes not aligned with our inclusion criteria; d. clinical trials that reported anemia as an adverse event; e. studies with purposive sampling and f. case series, case reports, commentaries, narrative and systematic reviews, non-peer reviewed studies, pre-prints and conference abstracts. In case of overlapping data between studies, the study with the largest dataset was retained. All retrieved studies were independently assessed by two reviewers VG, EMD) and any disagreements were resolved after discussion with the senior author (SG).

**Quality Control and Bias Assessment process**

Quality control and bias assessment was conducted independently by two reviewers (VG, EMD), and disagreements were settled by consensus after discussion with the senior author (SG). Data from individual studies, including author names, date of publication, study design, country, event type, and patient characteristics, were extracted in structured reports for further analyses.

**Statistical Analysis**

For the aggregate meta-analysis, we calculated for each dichotomous outcome of interest the corresponding pooled prevalence with 95% confidence intervals (95%CI), after the implementation of the Freeman-Tukey variance-stabilizing double arcsine transformation(16). The random-effects model of meta-analysis (DerSimonian and Laird) was used to calculate the pooled estimates(17). Pooled prevalence for the pre-defined outcome of interest was produced by the meta-prop function of R-meta. Pairwise comparisons were conducted between MS patients and the general population and were reported using risk ratios (RRs) and corresponding 95% confidence intervals (95% CIs). Subgroup differences were assessed by the Q test for subgroups.^16^ Heterogeneity was assessed with the I^2^ and Cochran Q statistics. For the qualitative interpretation of heterogeneity, I^2^ values>50% and values>75% were considered to represent substantial and considerable heterogeneity, respectively(18). The significance level for the Q statistic was set at 0.1. Publication bias across individual studies was graphically assessed when more than four studies were included in each analysis for the primary outcomes of interest, using funnel and radial plot inspection as well as the Egger’s linear regression test, and the equivalent z test for each pooled estimate with a two-tailed p value <0.05 was considered statistically significant(19). All statistical analyses and figure production were carried out using RStudio for ioS (RStudio 2023.12.1+402 (“Ocean Storm”)).

E-Figure 1

E-Figure 1 ROBINS-E traffic light plot. Low to moderate degree of bias mainly due to measurement and reporting of outcomes

E-figure 2

E-Figure 2 Funnel plot. Moderate bias without small study effects

E-table 1

| **Omitted Study** | **Pooled Effect** | **Lower CI** | **Upper CI** |
| --- | --- | --- | --- |
| Edwards et al.2004 | -1.990552 | -2.393214 | -1.587890 |
| Buchanan et al.2004 | -2.115586 | -2.587835 | -1.643338 |
| Kang et al.2010 | -2.033324 | -2.478821 | -1.587827 |
| Füvesi et al.2010 | -2.053254 | -2.510582 | -1.595925 |
| Marrie et al.2010 | -2.074964 | -2.542385 | -1.607542 |
| Rodrigo et al.2011 | -2.187478 | -2.628882 | -1.746074 |
| Miri et al.2013 | -2.183861 | -2.628943 | -1.738780 |
| Shaygannejad et al.2013 | -2.162566 | -2.620717 | -1.704416 |
| Koudriavtseva et al.2015 | -2.139520 | -2.606999 | -1.672041 |
| Tettey et al.2016 | -2.116118 | -2.587493 | -1.644743 |
| Cikrikcioglu et al.2016 | -2.130463 | -2.600241 | -1.660684 |
| Yusuf et al.2020 | -2.131313 | -2.601435 | -1.661192 |
| Lo et al.2020 | -2.120234 | -2.592134 | -1.648334 |
| Mendizabal et al.2020 | -2.098093 | -2.570199 | -1.625987 |
| Chou et al.2020 | -2.093319 | -2.564771 | -1.621867 |
| Afifi et al.2021 | -2.209448 | -2.630149 | -1.788746 |
| Bhattacharjee et al.2021 | -2.067674 | -2.531846 | -1.603503 |
| Maric et al.2021 | -2.053260 | -2.511649 | -1.594870 |
| Donze et al.2022 | -2.102998 | -2.574783 | -1.631212 |
| Padarti et al.2022 | -2.123155 | -2.594827 | -1.651482 |
| Li et al.2024 | -2.084037 | -2.553872 | -1.614202 |
